# Reasons for Electronic Cigarette Use Among Middle and High School Students — National Youth Tobacco Survey, United States, 2016

**DOI:** 10.15585/mmwr.mm6706a5

**Published:** 2018-02-16

**Authors:** James Tsai, Kimp Walton, Blair N. Coleman, Saida R. Sharapova, Sarah E. Johnson, Sara M. Kennedy, Ralph S. Caraballo

**Affiliations:** ^1^Office on Smoking and Health, National Center for Chronic Disease Prevention and Health Promotion, CDC; ^2^Office of Science, Center for Tobacco Products, Food and Drug Administration, Silver Spring, Maryland.

Electronic cigarettes (e-cigarettes) were the most commonly used tobacco product among U.S. middle school and high school students in 2016 (*1*). CDC and the Food and Drug Administration (FDA) analyzed data from the 2016 National Youth Tobacco Survey (NYTS) to assess self-reported reasons for e-cigarette use among U.S. middle school (grades 6–8) and high school (grades 9–12) student e-cigarette users. Among students who reported ever using e-cigarettes in 2016, the most commonly selected reasons for use were 1) use by “friend or family member” (39.0%); 2) availability of “flavors such as mint, candy, fruit, or chocolate” (31.0%); and 3) the belief that “they are less harmful than other forms of tobacco such as cigarettes” (17.1%). The least commonly selected reasons were 1) “they are easier to get than other tobacco products, such as cigarettes” (4.8%); 2) “they cost less than other tobacco products such as cigarettes” (3.2%); and 3) “famous people on TV or in movies use them” (1.5%). Availability of flavors as a reason for use was more commonly selected by high school users (32.3%) than by middle school users (26.8%). Efforts to prevent middle school and high school students from initiating the use of any tobacco product, including e-cigarettes, are important to reduce tobacco product use among U.S. youths (*2*).

NYTS is a school-based, pencil-and-paper questionnaire, self-administered to a cross-sectional, nationally representative sample of students in grades 6–12 in the United States (*3*). In 2016, 20,675 students completed the NYTS; the overall survey response rate was 71.6%. Reasons for e-cigarette use were assessed among both ever and current e-cigarette users. Ever users were defined as participants who responded “yes” to the question, “Have you ever used an electronic cigarette or e-cigarette, even once or twice?” Among ever users, current users were those who reported using e-cigarettes on ≥1 day during the past 30 days, based on responses to the question, “During the past 30 days, on how many days did you use electronic cigarettes or e-cigarettes?” Current e-cigarette users were further classified into four mutually exclusive group based on tobacco products used: e-cigarettes only; e-cigarettes and combustible tobacco (e.g., cigarettes, cigars, pipes, bidis, or hookah); e-cigarettes and noncombustible tobacco (e.g., smokeless tobacco, snus, or dissolvable tobacco); and e-cigarettes with combustible and noncombustible tobacco. Data for the group that used e-cigarettes and noncombustible tobacco products are not presented because of small sample size.

Participants were asked, “What are the reasons why you have used electronic cigarettes or e-cigarettes?” Response options were “I have never tried an electronic cigarette,” “friend or family member used them,” “to try to quit using tobacco products, such as cigarettes,” “they cost less than other tobacco products, such as cigarettes,” “they are easier to get than other tobacco products, such as cigarettes,” “famous people on TV or in movies use them,” “they are less harmful than other forms of tobacco, such as cigarettes,” “they are available in flavors, such as mint, candy, fruit, or chocolate,” “they can be used in areas where other tobacco products, such as cigarettes, are not allowed,” and “I used them for some other reason.” Participants could select multiple reasons.

After excluding participants who had never tried an e-cigarette or had missing information on school level (middle or high), sex, or race/ethnicity (non-Hispanic white, non-Hispanic black, Hispanic, and non-Hispanic other race), 4,049 ever users, including 1,281 current users, were included in the analysis. Data were weighted to account for the complex survey design and adjusted for nonresponse. Point estimates, 95% confidence intervals, and population totals corresponding to reasons for use were computed among ever and current e-cigarette users, both overall and by school level, sex, race/ethnicity, and current use of other tobacco products. Chi-square tests were used to assess statistically significant (p<0.05) differences across groups.

Among U.S. middle and high school e-cigarette ever users in 2016, the most commonly selected reasons for using e-cigarettes were “friend or family member used them” (39.0%), “they are available in flavors, such as mint, candy, fruit, or chocolate” (31.0%), and “they are less harmful than other forms of tobacco such as cigarettes” (17.1%). Reasons for use varied by school level and sex. For example, “friend or family member used them” was more commonly selected by middle school students (43.7%) than high school students (37.5%), and by females (46.7%) than males (32.2%) (Table 1). “They are available in flavors, such as mint, candy, fruit, or chocolate” was more commonly selected by high school students (32.3%) than by middle school students (26.8%). Among e-cigarette ever users, the least commonly selected reasons for use were “they are easier to get than other tobacco products, such as cigarettes” (4.8%), “they cost less than other tobacco products such as cigarettes” (3.2%), and “famous people on TV or in movies use them” (1.5%) (Table 1).

**TABLE 1:** Reasons for e-cigarette use among middle and high school students who reported ever using e-cigarettes by sex, race or ethnicity, and education level — National Youth Tobacco Survey, United States, 2016

Reason for e-cigarette use^†^	Ever used e-cigarettes*											
	Middle school users only (sample n = 1,061)		High school users only (sample n = 2,988)		Middle and high school users (sample n = 4,049)							
					Overall		Sex		Race/Ethnicity			
							Male	Female	White, non-Hispanic	Black, non-Hispanic	Hispanic^§^	Other race, non-Hispanic
	No. of users^¶^	% (95% CI)	No. of users	% (95% CI)	No. of users	% (95% CI)	% (95% CI)	% (95% CI)	% (95% CI)	% (95% CI)	% (95% CI)	% (95% CI)
Friend or family member used them	586,000	43.7** (40.5–46.9)	1,605,000	37.5** (35.3–39.8)	2,191,000	39.0 (37.4–40.6)	32.2** (30.1–34.2)	46.7** (44.0–49.5)	38.7 (36.0–41.4)	36.7 (31.2–42.6)	39.9 (36.3–43.6)	43.0 (34.7–51.7)
To try to quit using tobacco products such as cigarettes	―^††^	―^††^	372,000	8.7 (7.0–10.8)	440,000	7.8 (6.5–9.5)	8.7 (6.9–10.9)	6.8 (4.9–9.4)	10.0 (8.0–12.3)	―^††^	5.2 (3.8–7.0)	―^††^
They cost less than other tobacco products such as cigarettes	―^††^	―^††^	153,000	3.6 (2.9–4.4)	181,000	3.2 (2.6–3.9)	3.9 (2.9–5.2)	2.5 (1.8–3.4)	3.6 (2.9–4.5)	―^††^	―^††^	―^††^
They are easier to get than other tobacco products such as cigarettes	70,000	5.2 (3.7–7.3)	202,000	4.7 (3.8–5.9)	272,000	4.8 (4.1–5.8)	5.0 (3.9–6.2)	4.7 (3.8–5.8)	4.5 (3.6–5.6)	―^††^	5.9 (4.5–7.8)	―^††^
Famous people on TV or in movies use them	―^††^	―^††^	―^††^	―^††^	84,000	1.5 (1.0–2.2)	―^††^	―^††^	―^††^	―^††^	―^††^	―^††^
They are less harmful than other forms of tobacco, such as cigarettes	216,000	16.1 (13.7–18.9)	743,000	17.4 (15.5–19.4)	959,000	17.1 (15.6–18.7)	19.9** (17.6–22.4)	13.9** (11.5–16.6)	16.5 (14.5–18.7)	12.7 (10.4–15.5)	18.9 (16.4–21.7)	―^††^
They are available in flavors, such as mint, candy, fruit, or chocolate	360,000	26.8** (23.7–30.3)	1,382,000	32.3** (30.3–34.5)	174,000	31.0 (29.4–32.7)	31.4 (28.8–34.1)	30.6 (28.1–33.2)	29.0 (26.4–31.7)	33.5 (26.6–41.1)	34.6 (32.0–37.2)	32.1 (23.9–41.6)
They can be used in areas where other tobacco products, such as cigarettes are not allowed	―^††^	―^††^	337,000	7.9 (6.6–9.4)	393,000	7.0 (5.8–8.5)	7.2 (5.8–9.1)	6.7 (5.1–8.8)	7.3 (6.0–8.8)	―^††^	7.2 (4.8–10.7)	―^††^
Some other reason	422,000	31.5 (27.5–35.6)	1,351,000	31.6 (29.5–33.8)	1,773,000	31.6 (29.6–33.6)	31.5 (29.3–33.8)	31.6 (29.0–34.4)	32.1 (29.4–35.1)	29.4 (24.3–35.1)	30.9 (28.0–34.1)	31.8 (24.3–40.3)

**Abbreviation:** CI = confidence interval.

* Participants who responded “yes” to the question “Have you ever used an electronic cigarette or e-cigarette, even once or twice?”

^†^ Response to the question, “What are the reasons why you have used electronic cigarettes or e-cigarettes? (Check all that apply)” are not mutually exclusive.

^§^ Persons of Hispanic ethnicity can be of any race or combination of races.

^¶^ Estimated number of users based on sample weight.

** p-value <0.05 from chi-square test for difference in percentages within specified levels of school (middle or high), sex, or race/ethnicity.

^††^ Unstable estimate because subgroup size <50 or relative standard error >0.3. Chi-square test was not conducted.

Among U.S. middle and high school students who reported using e-cigarettes (e-cigarettes only, e-cigarettes with combustible tobacco only, and e-cigarettes with combustible and noncombustible tobacco) during the past 30 days, the most commonly selected reasons for e-cigarette use were “they are available in flavors, such as mint, candy, fruit, or chocolate” (41.1%, 46.0%, and 29.1%, respectively), “friend or family member used them” (35.1%, 26.6%, and 20.2%, respectively), and “they are less harmful than other forms of tobacco, such as cigarettes” (23.7%, 24.6%, and 22.8%, respectively) (Table 2).

**TABLE 2:** Reasons for e-cigarette use among middle and high school students who reported using e-cigarettes and other tobacco products during the past 30 days (current users) — National Youth Tobacco Survey, United States, 2016

Reason for e-cigarette use^†^	Use of e-cigarettes and other tobacco products during the past 30 days* (sample n = 1,281)					
	Use e-cigarettes only^§^ (sample n = 543)		Use e-cigarettes and combustible tobacco only ^¶^ (sample n = 419)		Use e-cigarettes with combustible and noncombustible tobacco (sample n = 273)	
	No. of users**	% (95% CI)	No. of users	% (95% CI)	No. of users	% (95% CI)
Friend or family member used them	276,000	35.1 (31.2–39.2)	157,000	26.6 (21.9–31.8)	85,000	20.2 (14.4–27.7)
To try to quit using tobacco products, such as cigarettes	―^††^	―^††^	109,000	18.5 (14.8–22.9)	110,000	26.3 (19.6–34.2)
They cost less than other tobacco products, such as cigarettes	―^††^	―^††^	―^††^	―^††^	―^††^	―^††^
They are easier to get than other tobacco products, such as cigarettes	―^††^	―^††^	―^††^	―^††^	―^††^	―^††^
Famous people on TV or in movies use them	―^††^	―^††^	―^††^	―^††^	―^††^	―^††^
They are less harmful than other forms of tobacco, such as cigarettes	187,000	23.7 (19.3–28.8)	145,000	24.6 (20.1–29.7)	95,000	22.8 (15.8–31.7)
They are available in flavors, such as mint, candy, fruit, or chocolate	324,000	41.1 (36.0–46.4)	271,000	46.0 (40.8–51.2)	122,000	29.1 (20.7–39.2)
They can be used in areas where other tobacco products, such as cigarettes, are not allowed	―^††^	―^††^	95,000	16.1 (12.8–20.2)	87,000	20.9 (15.4–27.6)
I used them for some other reason	270,000	34.3 (30.0–38.9)	199,000	33.8 (28.4–39.7)	124,000	29.7 (22.5–38.1)

**Abbreviation:** CI = confidence interval.

* Mutually exclusive categories. Subgroup for e-cigarettes and noncombustible tobacco (smokeless tobacco, snus, or dissolvable tobacco on ≥1 day in the past 30 days) is not shown because of small subgroup size.

^†^ Response to question, “What are the reasons why you have used electronic cigarettes or e-cigarettes? (Check all that apply)” are not mutually exclusive.

^§^ Reported use of only e-cigarettes on ≥1 day in the past 30 days.

^¶^ Reported use of e-cigarettes and only combustible tobacco including cigarettes, cigars, pipes, bidis, or hookah on ≥1 day in the past 30 days.

** Estimated number of users based on sample weight.

^††^ Unstable estimate because of subgroup size <50 or relative standard error >0.3.

## Discussion

Among U.S. middle and high school students who had ever used e-cigarettes in 2016, the most commonly selected reasons for e-cigarette use were “friend or family member used them,” “they are available in flavors, such as mint, candy, fruit, or chocolate,” and “they are less harmful than other forms of tobacco, such as cigarettes.” Regardless of whether users reported using e-cigarettes exclusively or with other tobacco products during the past 30 days, these reasons remained the most commonly selected reasons for e-cigarette use. The availability of flavors, use by a friend or family member, and belief that e-cigarettes are less harmful than other forms of tobacco might be important factors for initiation or maintenance of e-cigarette use among middle school and high school students. Although percentages reported here are lower, the findings from this study are consistent with those of previous studies reporting that availability of flavors is among the most prominently cited reasons for youths’ e-cigarette use (*4*,*5*).

The U.S. Surgeon General has concluded that e-cigarette use among youths and young adults is a public health concern (*2*). The prevalence of e-cigarette use among youths increased substantially during 2011–2015 (*6*,*7*). In 2016, e-cigarettes were the most common tobacco product used among adolescents, although the overall prevalence of use declined from previous years (*1*,*8*). The Surgeon General has also concluded that e-cigarettes can contain harmful and potentially harmful constituents, including nicotine (*2*,*8*); exposure to nicotine during adolescence can cause addiction and can harm the developing adolescent brain (*2*,*8*). Recent research indicated that e-cigarette use declined among adolescent students in 2016, likely in part because of population-based efforts to prevent youths’ e-cigarette initiation and use (*1*,*9*). Continued efforts are important to further reduce all forms of tobacco product use, including e-cigarettes, among U.S. youths. As noted by the Surgeon General, population-level strategies include incorporating e-cigarettes into smoke-free indoor air policies, restricting youths’ access to e-cigarettes in retail settings, licensing retailers, and establishing specific package requirements (*2*).

The findings in this report are subject to at least five limitations. First, because only students from public and private schools in the United States are recruited in the NYTS, the findings might not be generalizable to youths who are home-schooled, in detention centers, or have dropped out of school. Second, selected reasons for ever use of e-cigarettes might not necessarily be consistent with actual reasons for use in the 30 days before the survey. Third, self-reported data are subject to underreporting and recall bias (*10*). Fourth, only predetermined potential reasons were assessed, rather than participant-generated reasons for use. For example, 31.6% of ever users indicated “I used them for some other reason.” Thus, the importance of these reasons relative to other potential explanations cannot be assessed. Finally, the use of a response list, even with “select all that apply” available, might lead to underselection of other relevant reasons.

Comprehensive strategies to prevent and reduce the use of all tobacco products, including e-cigarettes, among U.S. youths are warranted (*2*). Regulation of the manufacturing, distribution, and marketing of tobacco products by FDA,* along with sustained implementation of comprehensive tobacco control and prevention strategies, could reduce youths’ e-cigarette initiation and use (*2*,*7*). In addition, continued monitoring of e-cigarette use, including reasons for use and product characteristics, is important to guide strategies to prevent and reduce use of e-cigarettes among youths.

**Summary**

**What is already known about this topic?**

Electronic cigarettes (e-cigarettes) were the most commonly used tobacco product among U.S. middle school and high school students in 2016. The Surgeon General concluded that e-cigarettes can contain harmful and potentially harmful constituents, including nicotine. Nicotine exposure during adolescence can cause addiction and can harm the developing adolescent brain.

**What is added by this report?**

Among student respondents to the National Youth Tobacco Survey reporting ever using e-cigarettes in 2016, the most commonly selected reasons for use were used by “friend or family member” (39%), availability of “flavors such as mint, candy, fruit, or chocolate” (31%), and the belief that “they are less harmful than other forms of tobacco such as cigarettes” (17%). The least commonly selected reasons were “they are easier to get than other tobacco products, such as cigarettes” (5%), “they cost less than other tobacco products such as cigarettes” (3%), and “famous people on TV or in movies use them” (2%).

**What are the implications for public health practice?**

Efforts to prevent middle school and high school students from initiating the use of any tobacco product, including e-cigarettes, are important to reduce tobacco product use among U.S. youths. Regulation of the manufacturing, distribution, and marketing of tobacco products by the Food and Drug Administration, along with sustained implementation of comprehensive tobacco control and prevention strategies, could reduce e-cigarette use and initiation by middle school and high school students.

## Footnotes

* https://www.federalregister.gov/articles/2016/05/10/2016-10685/deeming-tobacco-products-to-be-subject-to-the-federal-food-drug-and-cosmetic-act-as-amended-by-the.
